# Expression of the Longest *RGS4* Splice Variant in the Prefrontal Cortex Is Associated with Single Nucleotide Polymorphisms in Schizophrenia Patients

**DOI:** 10.3389/fpsyt.2016.00026

**Published:** 2016-02-29

**Authors:** Lan Ding, Miroslav Styblo, Zuzana Drobná, Ashok N. Hegde

**Affiliations:** ^1^Department of Nutrition, University of North Carolina at Chapel Hill, Chapel Hill, NC, USA; ^2^Department of Neurobiology and Anatomy, Wake Forest University School of Medicine, Winston-Salem, NC, USA; ^3^Department of Biological and Environmental Sciences, Georgia College and State University, Milledgeville, GA, USA

**Keywords:** genotype, bipolar disorder, psychiatric, allele, postmortem, regulatory region

## Abstract

The *Regulator of G protein signaling 4* (*RGS4*) gene is a candidate susceptibility gene for schizophrenia (SCZ). Previous studies showed that the mRNA level of the longest splice variant *RGS4-1* was decreased in the dorsolateral prefrontal cortex (DLPFC) of SCZ patients compared with healthy controls. In this pilot study, we examined the possible mechanisms of *RGS4-1* mRNA reduction in SCZ. We genotyped SNP1 (rs10917670), rs2661347, SNP4 (rs951436), SNP7 (rs951439), SNP18 (rs2661319), and rs10799897 (SNP9897) and tested the methylation status of CpG islands of the *RGS4* gene in the postmortem DLPFC samples obtained from subjects with SCZ and bipolar disorder as well as healthy controls. *RGS4-1* mRNA level was associated with five SNPs (SNP1, rs2661347, SNP4, SNP7, and SNP18) and their haplotypes but not with SNP9897. In addition, this study revealed that *RGS4-1* mRNA was low in subjects with specific genotypes of SNP1, rs2661347, SNP4, SNP7, and SNP18. Lower *RGS4-1* mRNA expression in the DLPFC of SCZ is associated with SNPs in the 5′ regulatory region of the *RGS4* gene but not with the methylation status of its CpG islands.

## Introduction

The *Regulator of G protein signaling 4* (*RGS4*) gene is associated with susceptibility to schizophrenia (SCZ). A previous microarray study showed that *RGS4* mRNA levels were decreased in prefrontal cortex (PFC) of SCZ patients as compared to healthy subjects (1). The four common single-nucleotide polymorphisms (SNP1, 4, 7, and 18) of the *RGS4* gene, localized between the 5′ upstream sequence and the second intron, as well as two haplotypes derived from these SNPs may confer SCZ risk (2). Some other SNPs in the 20 kb genomic region of *RGS4* are associated with SCZ (2), and the G-allele of SNP1 is associated with non-deficit SCZ (3). Other linkage and association studies also indicated a connection between *RGS4* polymorphisms and SCZ risk (4–6). *RGS4* polymorphisms (SNP1, 4, 7, and 18) were associated with differences in the dorsolateral prefrontal cortex (DLPFC) volume in first episode SCZ patients (7). Several *RGS4* SNPs are associated with clinical symptoms of SCZ. For example, Positive and Negative Symptoms Scale (PANSS) total and global psychopathology scores were associated with SNP4 (8). SNP18 (rs2661319) and rs2842030 were associated with more severe baseline PANSS total score (9). *A*t the baseline status, the A/A genotype at SNP7 of *RGS4* was associated with a poorer social function compared with the G/G genotype (10). SNP1 (rs10917670) was associated with the depression factor in SCZ (11).

We previously cloned five splice variants of *RGS4* mRNA expressed in the DLPFC of the human brain and three splice variants of *Rgs4* from the mouse brain and found that the transcriptional regulation of human *RGS4* differs from that of the mouse *Rgs4*. Our data showed that mouse *Rgs4* encodes one protein while human *RGS4* encodes four different proteins, three of which are unique to humans (12). We also found that the mRNA level of the longest isoform *RGS4-3* was specifically deceased in DLPFC of SCZ patients but not in healthy controls (NC) or subjects with bipolar disorder (BPD) (13). Since then, *RGS4-3* has been renamed *RGS4-1* (NM_001102445.2), and we use the new nomenclature here.

How is *RGS4-1* decreased in DLPFC of SCZ? One possibility is through epigenetic regulatory mechanisms such as DNA hypermethylation. Bioinfomatics analysis found CpG islands (GC% >50%, >200 bp and the ratio of observed/expected CpG >0.6) (14) spanning 246 bp located in the first intron and second exon of the *RGS4* gene. Hypermethylation of CpG islands leads to decrease in gene expression (15, 16), which might explain the reduction in *RGS4-1* mRNA level. To test whether the decrease in *RGS4-1* mRNA expression in the DLPFC of postmortem SCZ brains is due to epigenetic mechanisms, such as DNA hypermethylation, we investigated the methylation status of CpG islands of *RGS4*.

*RGS4-1* might also be decreased through mechanisms in which the regulatory region of the *RGS4* gene plays a role. Such a possibility might be revealed by testing whether the SNPs in the regulatory region of the *RGS4* gene are associated with *RGS4* mRNA expression. Therefore, we genotyped a few SNPs in the regulatory region of the *RGS4* gene including SNP1 (rs10917670), rs2661347, SNP4 (rs951436), SNP7 (rs951439), SNP18 (rs2661319), and rs10799897 (SNP9897) to test for possible associations between the SNPs and *RGS4* mRNA expression. We present results showing that expression of *RGS4-1* is associated with five SNPs and that *RGS4-1* expression is not correlated with the methylation status of CpG islands in the *RGS4* gene.

## Materials and Methods

### RNA and DNA Samples

RNA samples (RNA Array Collection) from the DLPFC of postmortem brains of the patients with SCZ, BPD, and normal controls (NC) (*N* = 35 in each group) were obtained from Stanly Medical Research Institute (SMRI) (see Tables S1 and S2 in Supplementary Material for demographic and other details), and mRNA expression levels in these samples were measured by quantitative real-time PCR (qPCR) (13). The corresponding DNA samples from the DLPFC and occipital cortex from the postmortem brains of these patients and NC were also obtained from SMRI.

The samples used in this study were all from post-mortem brain tissue, and therefore a protocol approved by the Institutional Review Board was not necessary as per institutional guidelines.

### SNP Genotyping Assays and Haplotype Determination

We used pre-designed Taqman SNP genotyping assays for SNP18 (rs2661319) and SNP9897 from Applied Biosystems (AB). Custom Taqman SNP genotyping assays for SNP4 (rs951436) and SNP7 (rs951439) (Table S3 in Supplementary Material) were designed using AB web site.^1^ We chose these SNPs because of their known association with SCZ. All the four SNPs were identified at the Genotyping Core of University of North Carolina (UNC) Chapel Hill. To verify the genotyping accuracy, 12 randomly selected samples were also genotyped again by Roche Light Cycler 480 using the same TaqMan assays. The two genotyping results completely match. Primers and probe sequences used for genotyping are shown in Table S4 in Supplementary Material. SNP1 (rs10917670) was identified by PCR and sequencing since it did not comply with the ABI custom Taqman assay design requirement. All analyses were carried out blind with respect to diagnosis. Haploview 4.2 (Broad Institute of MIT and Harvard)^2^ was used to perform Linkage Disequilibrium (LD) analysis. Based on population genotype, the program PHASE^3^ was used to reconstruct haplotype and assign diplotype for each individual and calculate haplotype frequency (17).

### Bioinformatics Analysis, Primer Design, and DNA Methylation Analysis

To identify possible CpG islands in the regulatory region and introns of the *RGS4* gene, a 3.2-kb putative promoter region including the 5′ regulatory region upstream of the transcription start site (TSS), exon1, 2 and intron 1 of *RGS4* were searched, and a 246 bp region containing nine CpG islands was found. The 105 DNA samples from SMRI were treated with bisulfite, and then used for PCR amplification and PCR products sequencing (18). Briefly, 125 ng of DNA was treated by bisulfite using EZ DNA methylation kit (Zymo Research Corporation, Irvine, CA, USA). Human Methylated and Non-Methylated DNA Set (Human HCT116 DKO Methylated and non-methylated DNAs) were used as positive and negative controls (Zymo Research Corporation). The bisulfite PCR primers for DNA methylation analysis of RGS4 were designed by using MethPrimer.^4^

The primers were: *RGS4*-BSP_fd 5′ TAGAGGGAGATAGAGGAGTTGGTATT 3′ and RGS4-BSP_rev 5′ ACAAACCTACAAACCCTTTACACAT3′. ZymoTaq™ DNA Polymerase was used to amplify bisulfite-treated DNA.

### Statistical Analysis

SAS software 9.2 (SAS Institute Inc., Cary, NC, USA) was used to perform statistical analysis. Pearson’s Correlation analysis was performed to identify possible correlations between the mRNA expression levels of two different splice variants. The association analysis of SNPs with SCZ or BPD was performed by using Haploview and PROC LOGISTIC regression analysis in the SAS software. The general linear model was used to analyze the relationship of the mRNA expression level with SNPs by using SAS software. When analyzing the association of genotypes with mRNA expression level of *RGS4* splice variants, an additive model was used, in which the phenotype of heterozygotes is intermediate to those of the two homozygotes. Previously, we found that the mRNA expression level of *RGS4-1* was significantly decreased in the DLPFC of SCZ compared to normal controls and was correlated with brain weight, and mRNA expression level of *RGS4-2* was correlated with brain pH (13). We did not, however, find a correlation between mRNA expression of *RGS4* isoforms and RNA integrity number (RIN), age, gender, postmortem interval, race, refrigerator interval, age of onset and duration of illness, lifetime use of alcohol, or antipsychotics. Therefore, to compare the effect of genotype or diplotype on mRNA level of *RGS4-1* in among all groups (including SCZ, NC, and BP groups), univariate analysis of covariance (ANCOVA) was performed, in which mRNA expression of *RGS4-1* or *RGS4-2* was a dependent variable, while genotype or diplotype was used as an independent factor and brain weight or brain pH as a covariate. To detect a possible interaction between SNP genotype and groups, SNP genotype and group were taken as two factors, and brain weight as a covariate.

When analyzing the relationship of SNPs with mRNA expression level of *RGS4-1* between groups, group (SCZ, BPD, or NC) was an independent variable, brain weight was a covariate, and *post hoc* test was used to identify the differences in *RGS4-1* mRNA levels between different groups. To determine the possible differences in the ratio between two splice variants among groups, general linear model was used, with the ratio as a dependent factor and the group as independent variable. To test whether the SNPs were related to ratios, the ratio was taken as a dependent variable and the genotype of SNPs and group were two factorial independent variables. Then *post hoc* Bonferroni test was applied.

## Results

### Association of mRNA Expression of RGS4 Splice Variants with RGS4 SNPs

Although we successfully genotyped six SNPs in the 5′ regulatory region and introns of *RGS4* in all samples from SMRI (see Table S1 in Supplementary Material for demographics), some RNA samples had to be excluded because of insufficient quantity and other limitations (reasons for which are detailed under Table S2 in Supplementary Material). Therefore, statistical analysis of the association between SNPs and the mRNA expression levels of *RGS4* splice variants was performed on samples from 27 individuals in each group (13). All DNA samples were from Caucasian subjects except for one from a Hispanic subject in the SCZ group and another from a Native American subject in the BPD group. Regardless of whether these two cases were included or not in the analysis, the statistical outcome remained the same. The mRNA expression levels of the isoforms *RGS4-2* to *RGS4-5* were not associated (*p* > 0.05) with the six SNPs of the *RGS4* gene that we examined. However, the mRNA expression of the longest isoform *RGS4*-1 was associated with the five SNPs (SNP1, rs2661347, SNP4, SNP7 and SNP18, *p* < 0.05) but not with SNP9897 (*p* > 0.05).

When the three groups were considered together including all samples from SCZ, normal controls (NC), and BPD, *RGS4-1* mRNA expression level was associated with the genotype of SNP1 [*F*(3,77) = 4.20, overall *p* = 0.0083, SNP1 genotype *p* = 0.0263] and rs2661347 [*F*(3,77) = 4.69, overall *p* = 0.0046, rs2661347 genotype *p* = 0.0141]. The mRNA expression levels of *RGS4-1* in the carriers with homozygous genotype TT of SNP1 or with allele T of SNP1 were lower than that from subjects with genotype CC (adjusted *p* = 0.0220) or allele C of SNP1 (adjusted *p* = 0.0338). The genotype TT of rs2661347 or allele T of rs2661347 was associated with lower amounts of *RGS4-1* transcript compared with genotype AA of rs2661347 (*p* = 0.0117) (Figure 2) or allele A (*p* = 0.0106). *RGS4-1* mRNA expression level was also associated with the genotype of SNP4 [*F*(3,77) = 4.93, overall *p* = 0.0035, SNP4 genotype *p* = 0.0104], SNP7 [*F* (3,77) = 4.20, overall *p* = 0.0083, SNP7 genotype *p* = 0.0263], and SNP18 [*F* (3,77) = 3.86 overall *p* = 0.0126, SNP18 genotype *p* = 0.0413]. The genotype AA or allele A of SNP4 had lower mRNA of *RGS4-1* compared to TT (*p* = 0.0081) or allele T (*p* = 0.0106) (Figure 4). The genotype TT (*p* = 0.0220) or allele T (*p* = 0.0338) of SNP7 (Figure 4) and genotype AA (*p* = 0.0401) or allele A (*p* = 0.0267) of SNP18 were associated with lower *RGS4-1* mRNA expression level compared to CC or allele C of SNP7, or GG or G of SNP18 (Figure 6).

**Figure 1:**
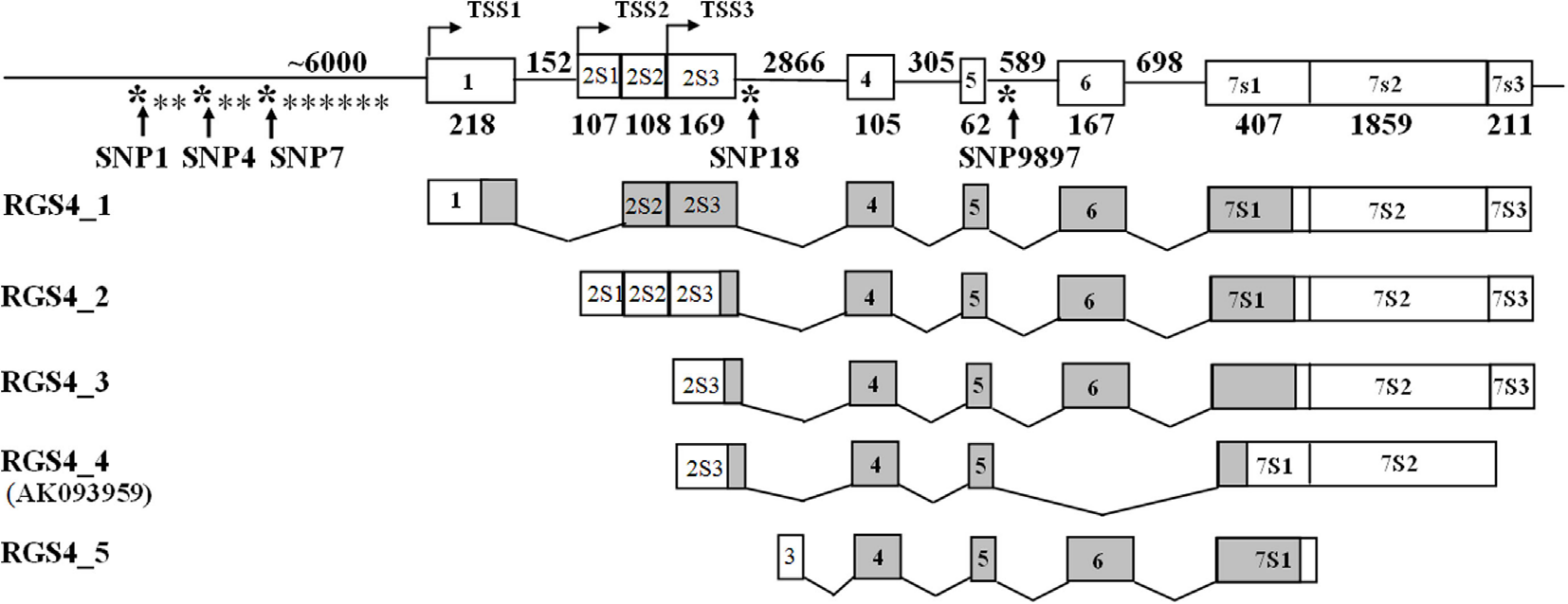
**Location of SNPs in the *RGS4* gene**. SNP1, SNP4, and SNP7 are located in the 5′ regulatory region of *RGS4*. SNP18 is in the second intron and SNP9897 is in the fourth intron of the *RGS4* gene.

**Figure 2:**
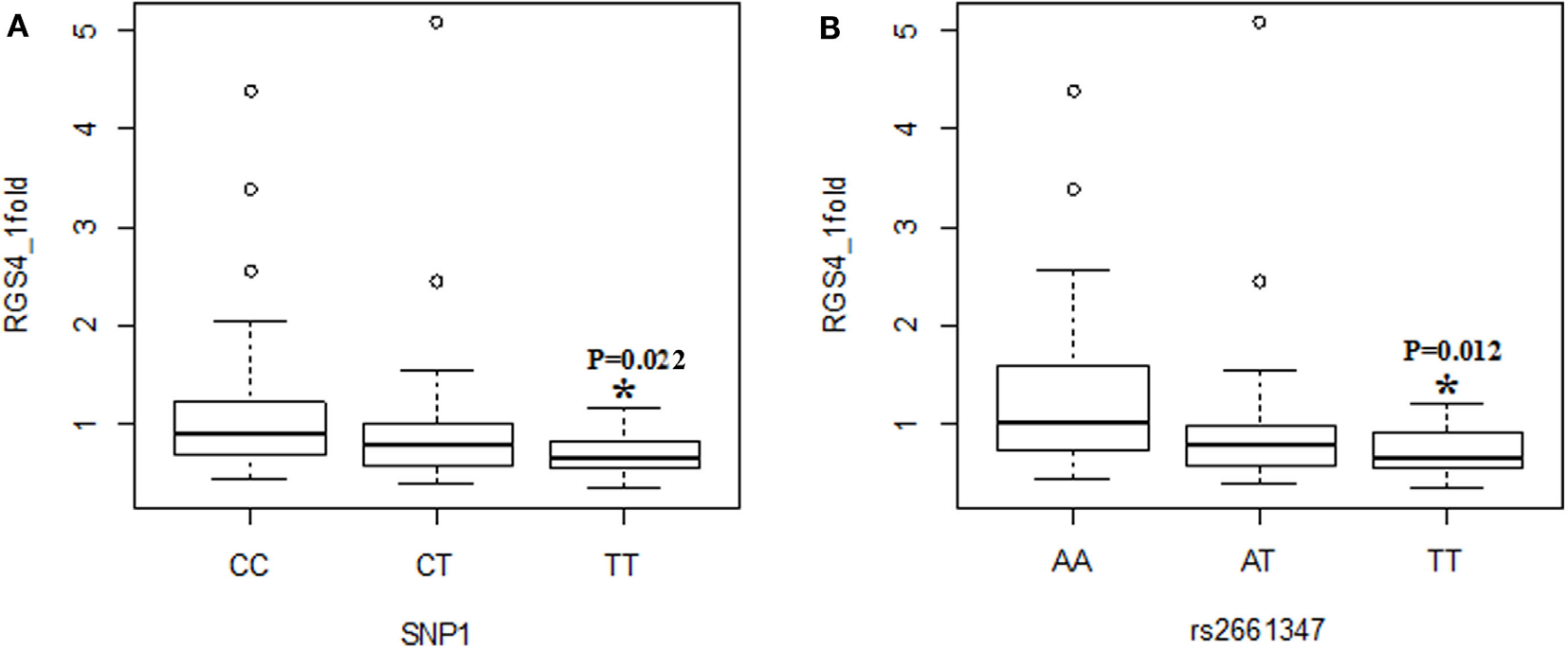
**Effects of SNP1 and rs2661347 on *RGS4-1* mRNA expression levels**. *RGS4-1* mRNA expression level in genotype TT of SNP1 was significantly lower than that of the genotype CC [*F*(3,77) = 4.20, overall *p* = 0.0083, *SNP1 genotype p* = 0.0263] **(A)**. The genotype TT of rs2661347 had significantly lower expression level of *RGS4-1* mRNA compared to the genotype AA [*F*(3,77) = 4.69, overall *p* = *0.0046*, rs2661347 genotype *p* = 0.0141] **(B)**.

**Figure 3:**
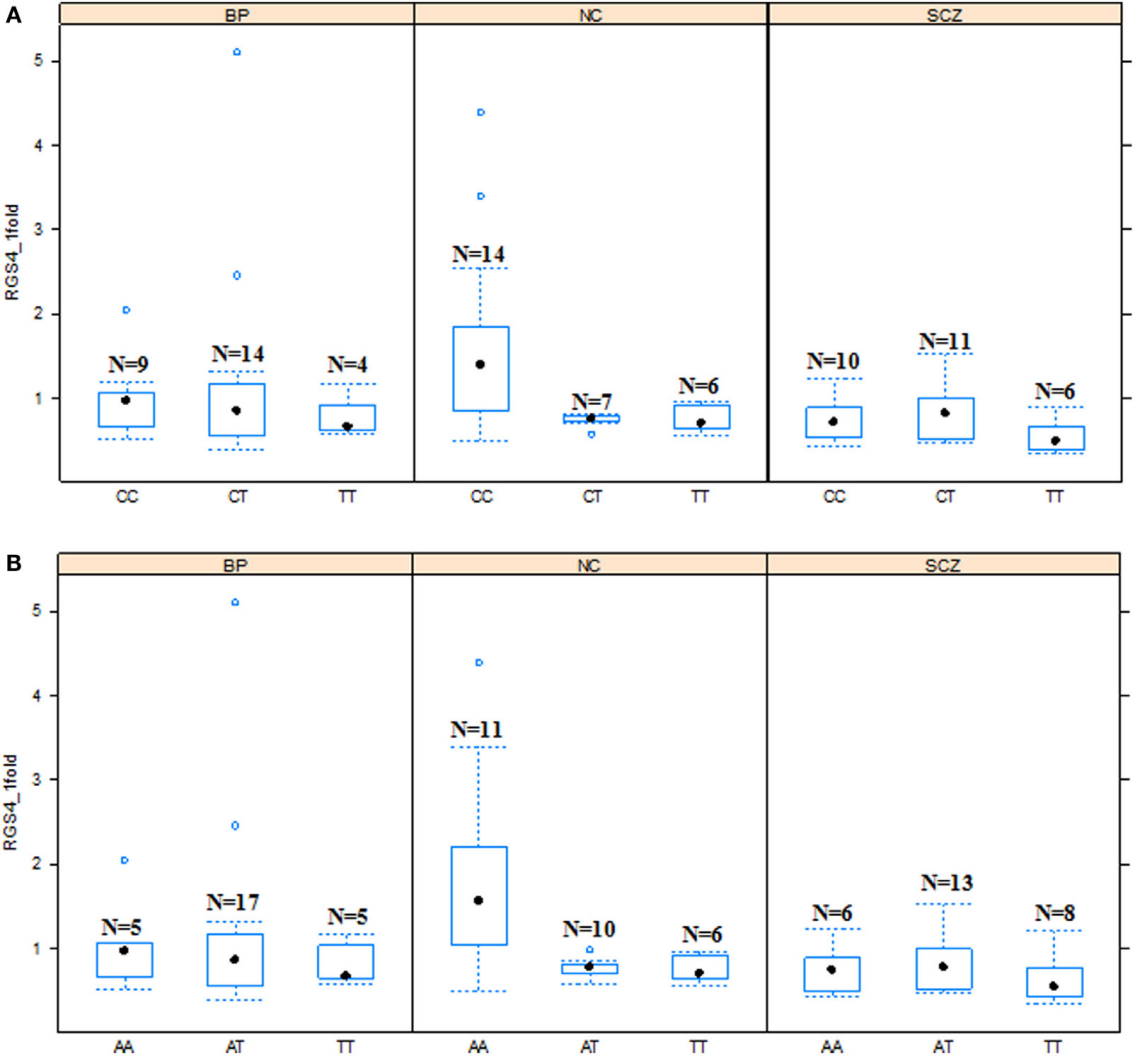
**Effects of SNP1 and rs2661347 genotypes on *RGS4-1* mRNA expression among the groups**. In the schizophrenia group (SCZ), the *RGS4-1* mRNA expression level in the subjects with genotype CC of SNP1 were lower than the people in the normal control (NC) group with same genotype CC (adjusted *p* = 0.0324) of SNP1 **(A)**. In the SCZ group, the patients with the genotype AA of rs2661347 (*p* = 0.0984) tended to have lower mRNA expression than those with the same genotype in the NC group **(B)**. Boxes represent 25th and 75th percentile distribution. Bars outside the box represent the SD.

**Figure 4:**
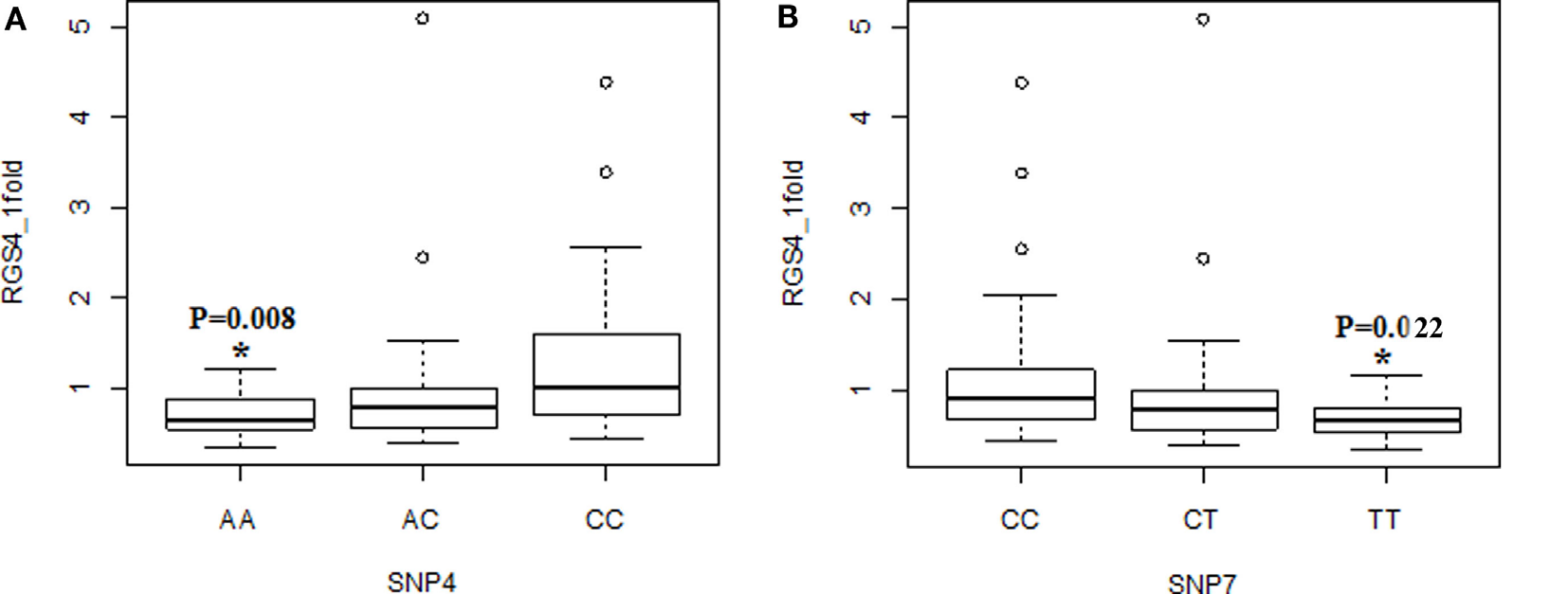
**Association of *RGS4-1* mRNA expression levels with the genotypes of SNP4 and SNP7**. The genotype AA of SNP4 was associated with the lower expression level of *RGS4-1*, and CC was related with the higher expression of RGS4-1 [*F*(3,77) = 4.93, overall *p* = 0.0035, *SNP4 genotype p* = 0.0104] **(A)**. The genotype TT of SNP7 was associated with the lower expression level, and CC with higher expression level of *RGS4-1* [*F*(3,77) = 4.20, overall *p* = 0.0083, SNP7 genotype *p* = 0.0263] **(B)**.

**Figure 5:**
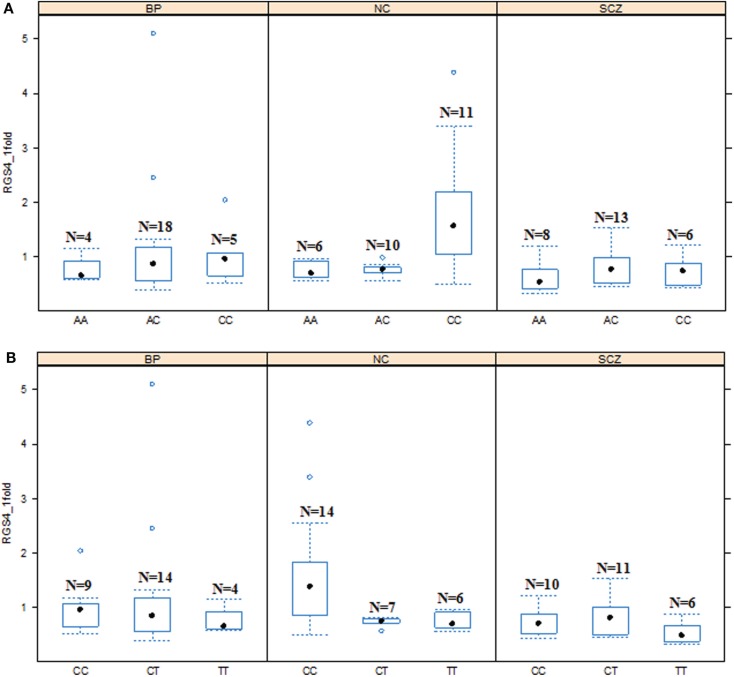
**Effects of SNP4 and SNP7 genotypes on *RGS4-1* mRNA expression among the groups**. **(A)**. In the schizophrenia (SCZ) group, the subjects with genotype CC of SNP4 tended to have lower mRNA expression level of *RGS4-1* than the subjects with the same genotype CC of SNP4 in NC (*p* = 0.0984) **(B)**. The mRNA of *RGS4-1* in SCZ patients with genotype CC of SNP7 was lower than those of the individuals with the same genotype CC in NC (*p* = 0.0373).

**Figure 6:**
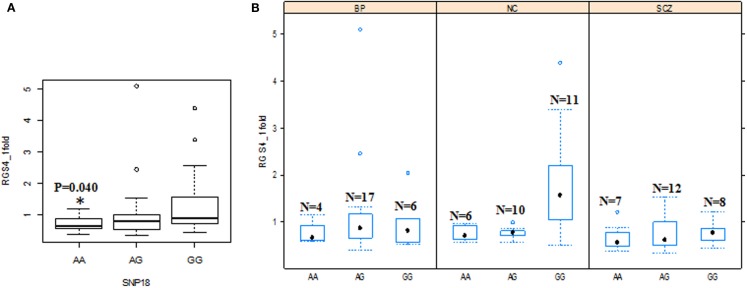
**Effects of SNP18 genotypes on *RGS4-1*mRNA expression**. **(A)** Among all the groups, the individuals with the genotype AA of SNP18 had lower mRNA expression of *RGS4-1* compared with the subjects with genotype GG of SNP18 [*F* (3,77) = 3.86 overall *p* = 0.0126; SNP18 genotype *p* = 0.0413]. **(B)** In SCZ, the subjects with genotype GG of SNP18 had lower mRNA expression level of *RGS4-1* compared with the subjects with the same genotype GG of SNP18 in NC (*p* = 0.0393).

In addition, in the univariate ANCOVA model to find the risk factors for decreased *RGS4-1* mRNA level, the interaction of SNP18 genotype × group (SCZ/BPD/NC) was statistically significant (*p* < 0.05) and the interaction of rs2661347 and SNP4 with group was close to statistical significance. The *p*-values for interaction between the group and SNP1, rs2661347, SNP4, SNP7, or SNP18 were 0.1698, 0.0588, 0.0549, 0.1698, and 0.0347, respectively. When we compared the effects of these genotypes on the *RGS4-1* mRNA levels between groups, we found that in the SCZ group, the genotype CC of SNP1 was related to the lower expression level of *RGS4-1* in the DLPFC compared to the NC group (*p* = 0.0385, Figure 3). In SCZ, the subjects with the genotype AA of rs2661347 (*p* = 0.0984) or genotype CC of SNP4 (*p* = 0.0984) tend to have lower *RGS4-1* mRNA expression level in the DLPFC than those with the same genotype in the NC group (Figures 3 and 5). In the SCZ group, the samples from patients with genotype CC of SNP7 (*p* = 0.0373) or the genotype GG of SNP18 (*p* = 0.0393) were associated with lower mRNA expression level of *RGS4-1* in the DLPFC than the subjects with the same genotype in the NC group (Figures 5 and 6).

To test whether expression of a given *RGS4* isoform is positively or negatively correlated with the expression of other *RGS4* isoforms, Pearson correlation analyses were performed, which showed that among different *RGS4* splice variants only the mRNA level of *RGS4-4* was positively correlated with that of *RGS4-5*. When mRNA expression of all *RGS4* isoforms were quantified collectively using a set of primers that detect all five *RGS4* isoforms [referred to as *Pan-RGS4* in our previous study (13)] as expected, we found that *Pan-RGS4* expression was positively correlated with expression of all *RGS4* isoforms.

### Linkage Disequilibrium Analysis of SNPs and the Haplotypes of RGS4 SNPs

We carried out linkage disequilibrium (LD) analysis of *RGS4* SNPs SNP1, rs2661347, SNP4, SNP7, rs2661319, and SNP9897 in all subjects including the SCZ, NC, and BPD groups. We found that SNP1, rs2661347, SNP4, and SNP7, were in disequilibrium with each other and were haplotype tagging SNPs (Figure 8).

**Figure 7:**
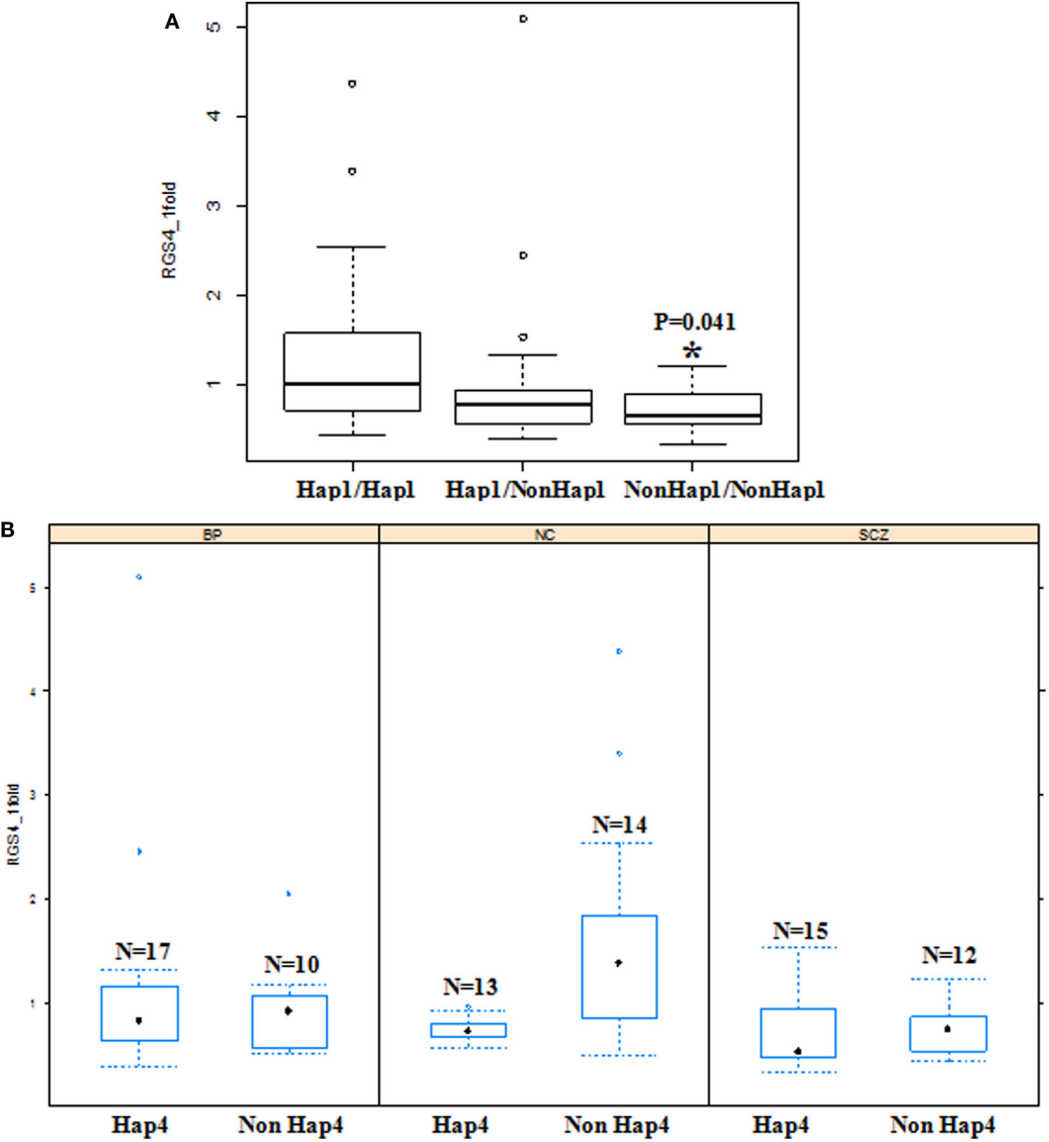
**Effects of haplotypes on *RGS4-1* mRNA expression**. **(A)** Effect of haplotype 1(Hap1) on the mRNA expression of *RGS4-1*. The individuals with diplotype of two non-Hap1 have lower *RGS4-1* mRNA expression level (*p* = 0.0413). **(B)** In the SCZ group, non-Hap4 carriers have lower *RGS4-1* mRNA expression level compared to those with non-Hap4 in NC (*p* = 0.0192). Hap1 is CACCG, Hap4 is TTATA.

**Figure 8:**
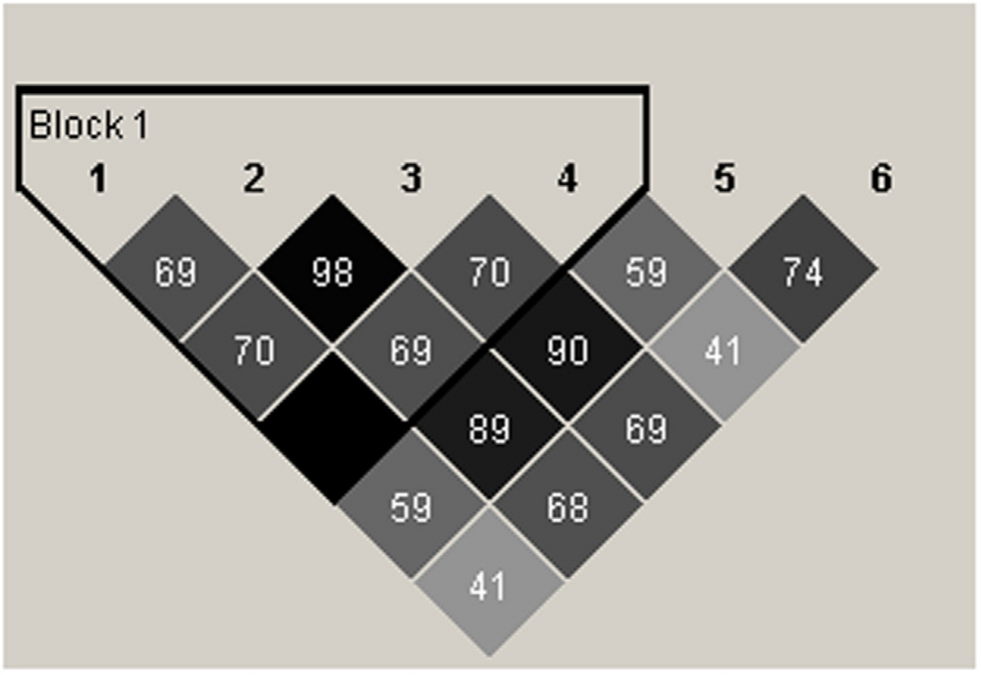
**Schematic representation of linkage disequilibrium (LD) between *RGS4* SNPs in the SCZ, BPD, and NC groups**. The LD block (thick black outline) consisting of four SNPs spans <1 kb (hence the Haploview program shows it as 0 kb). The number shown in each box in the figure corresponds to *r*^2^ which measures LD. *r*^2^ Values were calculated from the genotype data from the entire samples by Haploview. 1 = rs10917670 (SNP1), 2 = rs2661347, 3 = rs951436(SNP4), 4 = rs951439(SNP7), 5 = rs2661319(SNP18), 6 = rs10799897.

The constructed haplotypes and their frequencies obtained from the program PHASE in the three groups are shown in Table S5 in Supplementary Material. There are five haplotypes; the most common haplotype is hap1 (CACCG), and the second frequent one is hap4 (TTATA).

### Association between Haplotypes of RGS4 SNPs and the mRNA Expression of RGS4 Splice Variants

The haplotypes of *RGS4* SNPs were not associated with the mRNA expression of *RGS4-2*, *RGS4-3*, *RGS4-4*, and *RGS4-5*. However, when all the groups were analyzed together as a whole, the diplotype (Hap1/Hap1) was associated with higher *RGS4-1* mRNA expression level, and Non-Hap1 diplotypes (Hap2Hap2, Hap2Hap3, Hap4Hap4, and Hap4Hap5) were associated with lower *RGS4-1* mRNA expression level [*F*(3,77) = 4.69 overall *p* = 0.0046, haplotype *p* = 0.0141]. Non-Hap1 carriers had lower *RGS4-1* mRNA expression level (*p* = 0.0117). In the SCZ group, Non-Hap4 diplotype (Hap1Hap2, Hap1Hap5, Hap2Hap2, Hap1Hap1, Hap2Hap3) or Non-Hap4 carrier had lower *RGS4-1* mRNA expression level compared to NC [*F*(3,32) = 3.57, overall *p* = 0.0246, Group *p* = 0.0171] (Figure 7).

### Altered Ratio of RGS4 Splice Variants in SCZ and BPD

Changes in ratios between splice variants could contribute to the development of some diseases. For example, in sporadic colorectal cancer, the ratio of splice variants K-ras 4A and 4B is altered (19). To examine whether the ratio of *RGS4* splice variants changes in the DLPFC of SCZ or BPD, the ratio between two splice variants (ratio 1 to 2) was calculated using the formula 2^(Ct2 − Ct1)^ where Ct1 is threshold Ct value of splice variant 1, and Ct2 is threshold Ct value of splice variant 2 as measured by qPCR. The ratio of the mRNA expression level of *RGS4-1* to *RGS4-3* were different among groups [*F*(2,78) = 3.78, overall *p* = 0.0270]. The ratio decreased in SCZ (mean ratio = 0.0310) compared to NC (mean ratio = 0.0525) (*p* = 0.0237). The ratios of *RGS4-1* to other isoforms were not significantly different (*p* > 0.05). The ratio of mRNA expression level of *RGS4-2* and *RGS4-3* decreased in BPD (mean ratio = 0.0048) compared to NC (mean ratio = 0.0069) [*F*(2,78) = 4.58, overall *p* = 0.0131, *p* = 0.0114 between BPD and NC]. The ratio of mRNA expression level of *RGS4-2* to *RGS4-5* was different among the three groups [*F*(2,78) = 4.38, overall *p* = 0.0157]. Pairwise comparison test indicated that the ratio of *RGS4-2* to *RGS4-5* decreased in BPD (mean ratio = 5.63) compared to NC (mean ration = 8.81) (*p* = 0.0321), or compared to SCZ (mean ratio = 8.69) (*p* = 0.0426). The ratios of mRNA expression level of *RGS4-2* to other isoforms were not different. None of the SNPs was found to be associated with any ratio change of *RGS4* transcripts.

### SNP rs2661347 Genotype and Allele Frequency

Even though there have been detailed studies of *RGS4* SNPs 1, 4, 7, and 18 with respect to genotype and allele frequency, SNP rs2661347 is less well studied. Therefore, we calculated genotype and allele frequency of rs2661347 in 35 normal control Caucasian subjects from SMRI. The major allele (A) frequency is 0.557 (39/70), the frequency of minor allele (T) is 0.443 (31/70). The genotype frequency is A/A (0.343), A/T (0.429), and T/T (0.228). From the NCBI SNP database, in European population, the frequency of minor allele (A) of rs2661347 is 0.419 and that of major allele (T) is 0.581, and the genotype frequency is A/A (0.161), A/T (0.516), and T/T (0.323).^5^

### SNPs of the RGS4 and Transcription Factor Binding Sites

Bioinformatics analysis found that SNPs 4, 7, and 18 are located in the binding sites for transcription factors within the putative promoter region of *RGS4*. The transcription factor binding sites in the regulatory sequence surrounding the SNPs were searched by using MatInspector in Genomatix.^6^ SNP4 is located in the predicted binding sites of Onecut homedomain factor hepatocyte nuclear factor-6 (HNF6) (matrix similarity was 0.960) and LEF1/TCF (Matrix similarity was 0.887). The transcription factor HNF6 is a transcriptional activator, which controls the expression of transcription factors and is expressed at early stages of liver and in neuronal differentiation (20). LEF1/TCF is a transcription factor in the Wnt signaling pathway, which functions by recruiting the co-activator beta-catenin to the enhancer elements of target genes (21). LEF1/TCF is expressed in the hippocampus and is known to regulate the generation of dentate gyrus granule cells (22). LEF1/TCF together with beta-catenin activates genes that play a role in the proliferation and differentiation of neuronal precursor cells. These transcription factors also regulate transcription of the Cav3.1 calcium channel gene in thalamic neurons of the adult mouse brain (23).

SNP7 is located in the binding sites of zinc finger protein of the cerebellum (Matrix similarity was 0.894). SNP18 is located in the binding sites of mouse Krüppel-like factor (Matrix similarity was 0.992) and nuclear factor of activated T-cells (NFAT) (Matrix similarity is 0.839). Zinc finger proteins such as Zac1 play a key role in the development of specific neuronal subsets in the cerebellum (24). Krüppel-like factor (KLF) such as KLF7 acts as a transcriptional activator and regulates development of dopaminergic neurons in the olfactory bulb (25). NFAT regulates transcription in NMDA receptor-stimulated cortical neurons (26).

### Absence of Hypermethylation in the CpG Islands of RGS4 in the DLPFC of SCZ Patients

To study the methylation status of CpG islands of *RGS4*, all the 105 DNA samples from the DLPFC of SCZ, NC, and BPD groups (35 in each group) from SMRI were bisulfite-treated, then PCR-amplified and the amplified DNA fragments were sequenced. In every experiment with bisulfite treatment and sequencing of PCR products, methylated and non-methylated human DNA samples were included as positive and negative controls, respectively. Sequencing results showed that all the methylated Cs in treated methylated DNA samples remained unchanged, whereas all the Cs changed to Ts in all the treated non-methylated DNA samples. However, no hypermethylation of CpG islands in the *RGS4* gene was found in these samples. Therefore, hypermethylation of CpG islands in the regulatory region of RGS4 does not appear to be a causative factor for decreased mRNA expression level of *RGS4-1* observed in the DLPFC of SCZ patients.

## Discussion

### Association of mRNA Expression of RGS4 Splice Variants with RGS4 SNPs

Previously, we found that the mRNA expression level of the longest *RGS4* isoform, *RGS4-3* (which is now *RGS4-1*, according to the new nomenclature) was decreased in the DLPFC of SCZ patients (13). In the present study, to test the possible link between *RGS4* SNPs and the decrease in *RGS4* splice variants, we investigated the relationships of the genotypes, alleles, and haplotypes of the SNPs with the mRNA levels of *RGS4* isoforms. Among the five isoforms, only *RGS4-1* mRNA levels were associated with these SNPs, but mRNA expression of other *RGS4* isoforms *(RGS4-2* to *RGS4-5)* were not. The four SNPs associated with *RGS4-1* mRNA levels are located in the 5′ regulatory region and therefore suggest a possible role for these SNPs in regulating *RGS4-1* mRNA expression, which may have broader implications for *RGS4* expression changes in other neuropsychiatric disorders (27) and drug addiction (28, 29). SNP18 is located in the longest intron of the *RGS4* gene and is in the binding site of the Krüppel-like transcription factor. Therefore, it is likely that this intron contains the regulatory elements that control *RGS4-1* mRNA expression.

We found that specific genotypes or alleles of SNPs of *RGS4* were associated with lower mRNA expression among the three groups. In the SCZ group, more individuals carry these risk alleles than in the NC group. The interaction of genotype and diagnosis was observed for SNP18. Some genotypes are associated with lower *RGS4-1* mRNA levels in the SCZ group compared to NC. These lines of evidence suggest that there might be a genetic explanation for our previous finding of lower *RGS4-1* mRNA level in the DLPFC of the SCZ group compared to the NC group (13). Since our sample size was not large, the results of our study need to be interpreted cautiously and additional future studies may be warranted. Nonetheless, our studies provide useful initial evidence for linking *RGS4* mRNA expression to its SNPs similar to other such studies (30, 31).

For a number of tests in this study, we used Bonferroni adjustment for pairwise comparison in each analysis. For example, to test the effect of SNP genotype on *RGS4-1* expression, mRNA level was used as dependent factor, SNP genotype as independent variable and brain weight as covariate, and Bonferroni adjustment *post hoc* test was applied. We did not, however, perform further Bonferroni correction for multiple testing. This is because the six tested SNPs of *RGS4* were not chosen randomly but were based on prior SNP association studies and their physical location in the 5′ UTR and in the putative regulatory intronic regions of *RGS4*. In addition, these SNPs were in LD and the degree of independence between them was low. Moreover, the five splice variants of *RGS4* are not independent. Therefore, further correction for random effects would be extremely conservative and would result in a high type II error rate. The approach employed by us has been used before to investigate the relationship of SNPs of another SCZ candidate gene, Neuregulin 1 (32).

Another study (33) showed that *RGS4* mRNA in the gray matter of DLPFC and the hippocampus in CBDB/NIMH collection and from Stanley collection did not change and none of the SNPs of *RGS4* had a significant effect on *RGS4* mRNA expression. As we discussed previously (13), judging by the PCR primers used in the Lipska et al. study, it appears that these authors detected combined mRNA expression of *RGS4-1* to *RGS4-4* but not that of *RGS4-5*. We, however, used *RGS4-1* specific primers to detect its mRNA expression and found that it was associated with five individual SNPs or their haplotype or diplotype.

Three of the *RGS4* SNPs (SNP4, SNP7 and SNP18) are located in the binding sites of transcriptional factors. It is possible that the SNP genotypes affect binding of transcription factors to the *RGS4* cis-regulatory elements and thus affect *RGS4-1* expression in DLPFC of SCZ. It is worth noting that the transcripts of other SCZ susceptibility genes such as PDLIM5, Neuregulin 1, and Neuregulin 3 were found to be associated with SNPs in their 5′ regulatory regions and introns (32, 34, 35). SNP4 and SNP7 are located around 6 kb upstream of the transcriptional start site 1 (TSS1) (Figure 1) and SNP18 is in intron 2 of *RGS4* gene, which is close to putative TSS3. It is not clear whether these SNPs reside in the core promoter regions of *RGS4*. Thus far, the core promoter regions, cis-regulatory elements and the mechanisms of transcriptional regulation of *RGS4* splice variants have not been identified clearly. Therefore, any functional studies on *RGS4* SNP variants must await full characterization of multiple *RGS4* promoters and other transcriptional regulatory mechanisms.

Others investigating Val158Met genotype of Catechol-*O*-Methyl transferase (COMT) found that the carriers of the Val allele have significantly lower mRNA level of *RGS4* than the subjects who were homozygous for the Met allele (33). Thus, polymorphism of other genes such as that of COMT likely affects *RGS4* mRNA expression. It would be interesting to check whether the Val allele of COMT is associated with mRNA expression of a specific *RGS4* splice variant.

### RGS4 and SCZ

The SNPs (SNP1, 4, 7, and 18) of *RGS4* are associated with the clinical symptoms and antipsychotic treatment response in SCZ (8–10). Although there are some reports on lack of association between *RGS4* and SCZ (36, 37), many studies have reported an association between *RGS4* and SCZ (38, 39) and thus on balance, overall evidence is in favor of *RGS4* as a susceptibility gene for SCZ. In this study, we found that the decrease of *RGS4-1* mRNA level was associated with a specific genotype or allele of the SNPs in the 5′ regulatory regions and intron 2 of the *RGS4* gene. Therefore, our findings support the notion that *RGS4* SNPs might play a role in the etiology of SCZ through their influence on *RGS4-1* mRNA expression.

How might *RGS4* play a role in etiology of SCZ? One possibility is that RGS4 regulates glutamate signaling (40–42). Our studies (Ding et al., unpublished) showed that mouse *Rgs4* regulates presynaptic calcium channels and synaptic transmission via its action on a G-protein interacting with metabotropic glutamate receptors type 2. Others have interaction of Rgs4 with signaling by metabotropic glutamate receptors type 5 (43). Mouse *Rgs4* has three different splice variants, which all encode the same protein containing 205 amino acids (aa). The human *RGS4*, however, has five splice variants of which *RGS4-2* and *RGS4-3* encode a 205 aa protein similar to the mouse Rgs4. *RGS4-1* shares the 205 aa region with the mouse Rgs4 but has extra 97 aa at the N-terminus. *RGS4-5*, on the other hand, lacks 18 N-terminal aa compared to the 205-aa protein encoded by *RGS4-2* and *RGS4-3* (12). Therefore, RGS4-1 protein might differentially regulate glutamate signaling compared to other RGS4 proteins.

*RGS4* may also influence dopamine signaling and thus contribute to SCZ pathology. *RGS4* mRNA expression is associated with cortical dopamine signaling (33). The RGS4 N-terminal region can inhibit dopamine D2 and D3 receptor signaling (44) similar to other RGSs such as RGS9 (45). Therefore, RGS4-1 protein with its longer N-terminus might inhibit D2 and D3 receptor signaling and a decrease in RGS4-1 protein in the DLPFC of SCZ patients might cause an increase in D2 and D3 receptor signaling compared to normal subjects.

### RGS4 and BPD

A previous case-control study (484 patients and 288 controls) showed significant association of SNP rs951436 with BPD thus supporting *RGS4* as a potential BPD susceptibility gene (46). In another study, case-control comparisons revealed no significant differences for individual SNPs (SNP1, 4, 7, 18) between control and BPD, but an omnibus test in Brazil suggested differences in the overall distribution of haplotypes of all four SNPs (5). Previously, we found a trend toward lower *RGS4-2* mRNA expression in the DLPFC of BPD patients (13). In the current study, however, we found that the mRNA ratio of *RGS4-2* to *RGS4-3* and that of *RGS4-2* to *RGS4-5* decreased in the BPD group compared to NC (*p* < 0.05). The ratio of *RGS4-2* to other isoforms did not change. In addition, none of the *RGS4* SNPs was found to be associated with mRNA level of *RGS4-2* or with the ratio of *RGS4-2* to *RGS4-3* or to *RGS4-5* (*p* > *0.05*). The decreases in ratios of *RGS4-2* to *RGS4-3* and to *RGS4-5* hint at the possibility that *RGS4* might have a role in the etiology of BPD.

## Conclusion

The main finding of the present study is that levels of *RG4-1* mRNA are associated with SNPs in the regulatory region of the *RGS4* gene. An additional finding is that the ratios of *RGS4-2* to *RGS4-3* and to *RGS4-5* are decreased in PFC of BPD subjects relative to normal controls. A limitation of the study is the relatively small sample size. Future studies with larger sample sizes would be necessary to confirm our observations.

## Author Contributions

LD carried out most of the experiments, analyzed the data, and wrote the manuscript. ZD carried out some of the experiments. MS supervised some of the experiments carried out at UNC Chapel Hill by LD and ZD and helped with data analysis. ANH conceived the original project, designed the overall research and experiments, supervised Dr. LD’s experiments carried out at Wake Forest University School of Medicine, helped with interpretation of data, and edited the manuscript.

## Conflict of Interest Statement

The authors declare that the research was conducted in the absence of any commercial or financial relationships that could be construed as a potential conflict of interest.

## Footnotes

^1^https://www5.appliedbiosystems.com/tools/cadt/

^2^http://www.broadinstitute.org/scientific-community/science/programs/medical-and-population-genetics/haploview/haploview

^3^http://stephenslab.uchicago.edu/software.html

^4^http://www.urogene.org/

^5^http://www.ncbi.nlm.nih.gov/SNP/snp_ss.cgi?ss=ss51505122

^6^http://www.genomatix.de
