# Supplementary material for: Expression of the Longest RGS4 Splice Variant in the Prefrontal Cortex Is Associated with Single Nucleotide Polymorphisms in Schizophrenia Patients
Source: Front Psychiatry. 2016 Feb 29;7:26. doi: 10.3389/fpsyt.2016.00026 (PMC4770186; doi:10.3389/fpsyt.2016.00026)
Supplement: Supplementary file 1 [file Table_1.pdf]

Supplementary Table 1. Demographics and tissue characteristics of the included samples from the Stanley Medical Research Institute Array collection.

	Schizophrenia	Normal	Bipolar Disorder
Age (years, Mean±SD)	44±8	44±7	45±12
Gender	18M 9F	21M 6F	13M 14F
Race	26W 1H	27W	27W
Suicide status	6Y 21N	N/A	12Y 15N
Refrigerator interval (h)	7±5	4±3	8±6
Postmortem interval (h)	32±16	29±13	36±19
Brain pH	6.5±0.3	6.6±0.3	6.5±0.3
RIN	8.5±0.6	8.3±0.7	8.1±1.1
Brain weight (g)	1447±114	1446±154	1410±140
Age of onset (years)	22±7	N/A	24±9
Duration of illness (years)	22±10	N/A	21±10
Smoking at TOD	16Y 4N 7U	7Y 8N 12U	11Y 5N 11U
Psychotic feature	27Y	N/A	16Y 9N 2U
Lifetime antipsychotics	27Y	N/A	17Y, 9N, 1U

M, Male, F, Female; W, White, H, Hispanic, NA, Native American; N, no, Y, yes, U, unknown, SD, standard deviation
