# Supplementary material for: Expression of the Longest RGS4 Splice Variant in the Prefrontal Cortex Is Associated with Single Nucleotide Polymorphisms in Schizophrenia Patients
Source: Front Psychiatry. 2016 Feb 29;7:26. doi: 10.3389/fpsyt.2016.00026 (PMC4770186; doi:10.3389/fpsyt.2016.00026)
Supplement: Supplementary file 2 [file Table_2.pdf]

Supplementary Table 2. Demographics and tissue characteristics of the excluded samples* from the Stanley Medical Research Institute (SMRI) Array collection.

	Schizophrenia	Normal	Bipolar Disorder
Age (years, Mean±SD)	39±10	45±9	48±5
Sex	8F	4M 4F	4M 3F
Race	8W	8W	6W 1B
Suicide status	1Y 7N	N/A	3Y 4N
Refrigerator interval (h)	4±2	3±2	18±18
Postmortem interval (h)	28±13	31±13	47±17
Brain pH	6.4±0.2	6.6±0.3	6.3±0.4
RIN	8.4±0.6	7.9±1.0	7.0±1.5
Brain weight (g)	1427±86	1438±137	1334±129
Age of onset (year)	19±4	N/A	31±10
Duration of illness (year)	20±10	N/A	17±6
Smoking at TOD	7Y 1U	2Y 1N 5U	4Y 3N
Psychotic feature	8Y	N/A	5Y 2N
Lifetime antipsychotics	8Y	N/A	5Y 2N

M, Male, F, Female; W, White, H, Hispanic, NA, Native American; N, no, Y, yes, U, unknown, SD, standard deviation

* These samples were excluded because: of loss of one sample during delivery, lack of sufficient RNA in the original sample obtained from SMRI, of the difference in Ct value higher than 0.5 between the replicates of the same sample, the samples were extreme outliers with respect to Ct value of endogenous controls and delta-Ct values of targets and the endogenous controls, and one was a case of CADASIL (cerebral autosomal dominant arteriopathy with subcortical infarcts and leukoencephalopathy).
