# Supplementary material for: Expression of the Longest RGS4 Splice Variant in the Prefrontal Cortex Is Associated with Single Nucleotide Polymorphisms in Schizophrenia Patients
Source: Front Psychiatry. 2016 Feb 29;7:26. doi: 10.3389/fpsyt.2016.00026 (PMC4770186; doi:10.3389/fpsyt.2016.00026)
Supplement: Supplementary file 3 [file Table_3.pdf]

Supplementary Table 3. Details of *RGS4* SNPs.

dbSNP ID	SNP name	Assembly	Genome Build	Contig	Contig position	Intermarker distance(bp)
rs10917670	SNP1	GRCh37.p5	37.3	NT_004487.19	14521484	0
rs2661347	1347	GRCh37.p5	37.3	NT_004487.19	14521486	1
rs951436	SNP4	GRCh37.p5	37.3	NT_004487.19	14521984	497
rs951439	SNP7	GRCh37.p5	37.3	NT_004487.19	14522333	348
rs2661319	SNP18	GRCh37.p5	37.3	NT_004487.19	14528419	6085
rs10799897	9897	GRCh37.p5	37.3	NT_004487.19	14531730	3310
