# Supplementary material for: Expression of the Longest RGS4 Splice Variant in the Prefrontal Cortex Is Associated with Single Nucleotide Polymorphisms in Schizophrenia Patients
Source: Front Psychiatry. 2016 Feb 29;7:26. doi: 10.3389/fpsyt.2016.00026 (PMC4770186; doi:10.3389/fpsyt.2016.00026)
Supplement: Supplementary file 4 [file Table_4.pdf]

Supplementary Table 4. The Primers and probes for genotyping and bisulfate specific PCR.

| Primers and reporters | Sequences |
|---|---|
| Taqman genotype SNP4_forward | 5'TCCAGCCCCATATCTTTGCTTTT3' |
| Taqman genotype SNP4_reverse | 5'TTTACTACAATGGCCTAGACATCAATGAG3' |
| Taqman genotype SNP4 reporter 1 | 5'AGTTCAGTTATTGATTTTTAG3' |
| Taqman genotype SNP4 reporter 2 | 5'TTCAGTTATTGAGTTTTAG3' |
| Taqman genotype SNP4_forward | 5'TCCTGCTGTGTGGCTGAATG3' |
| Taqman genotype SNP4_forward | 5'TGTTGACAATAGGCTTTAGAAGAACTGTT3' |
| Taqman genotype SNP7 reporter 1 | 5'TCTGCCCCCCAGTAGG3' |
| Taqman genotype SNP7 reporter 2 | 5'TCTGCCCCTCAGTAGG3' |
| PCR SNP1_forward | 5'ATGGCACAGAACAGGGGAAATA3' |
| PCR SNP1_reverse | 5'TAGCCCAGGATAGAAACAGCAGT3' |
