# Supplementary material for: Expression of the Longest RGS4 Splice Variant in the Prefrontal Cortex Is Associated with Single Nucleotide Polymorphisms in Schizophrenia Patients
Source: Front Psychiatry. 2016 Feb 29;7:26. doi: 10.3389/fpsyt.2016.00026 (PMC4770186; doi:10.3389/fpsyt.2016.00026)
Supplement: Supplementary file 5 [file Table_5.pdf]

Supplementary Table 5. Haplotype frequency from PHASE in three groups.

Group	SNP1	rs2661347	SNP4	SNP7	SNP18	Haplotype	Frequency		
							SCZ	NC	BPD
SCZ	C	A	C	C	G	Hap1	0.471		
	C	T	A	C	A	Hap2	0.114		
	T	T	A	T	A	Hap4	0.356		
	T	T	A	T	G	Hap5	0.058		
NC	C	A	C	C	G	Hap1		0.557	
	C	T	A	C	A	Hap2		0.057	
	T	T	A	T	A	Hap4		0.386	
BP	C	A	C	C	G	Hap1			0.500
	C	T	A	C	A	Hap2			0.088
	C	T	C	C	G	HaP3			0.015
	T	T	A	T	A	Hap4			0.382
	T	T	A	T	G	Hap5			0.015
